# Cross-Bonded Cable Circuits Identification Based on Deep Embedded Clustering of Sheath Current Sensing

**DOI:** 10.3390/s26051591

**Published:** 2026-03-03

**Authors:** Hang Wang, Zhi Li, Wenfang Ding, Jing Tu, Liqiang Wang, Jun Chen

**Affiliations:** 1 Hubei Engineering Research Center for Safety Monitoring of New Energy and Power Grid Equipment, Hubei University of Technology, Wuhan 430068, China; wanghang@whu.edu.cn (H.W.); 19972580567@163.com (Z.L.); wenfangding@163.com (W.D.); 2 State Grid Hubei Electric Power Company, State Grid Corporation of China, No. 1701, Jiefang Road, Jiangan District, Wuhan 437399, China; tujing_128@163.com; 3 Wuhan Huayuan Electrical Power Company, No. 74, Hanyang Road, Hanyang District, Wuhan 430014, China; 13986187056@139.com

**Keywords:** HV cable, sheath current, circuit identification, cross-bonding

## Abstract

Online identification of HV cable circuits is vital for routine inspection and maintenance, yet existing passive electromagnetic wave injection methods are limited to offline operations. To fill the gap and achieve the online identification of HV cable circuits, an online circuit identification methodology based on sheath current temporal characteristics and deep embedded clustering is proposed. First, an equivalent circuit model of the multi-circuit cross-bonded cable sheath was built to deduce the temporal similarity of sheath currents within the same circuit, establishing the identification criterion. Second, the robustness of the temporal similarity under various operating conditions was verified via simulation based on the Dynamic Time Warping (DTW) distance. Then, a combined model of Temporal Convolutional Network Autoencoder (TCN-AE) and K-medoids was established to transform circuit identification into a temporal clustering problem of sheath currents, realizing circuit determination by synchronously monitoring the time-series sheath current data of multi-circuit HV cross-bonded cables. The method was verified on a full-scale 110 kV cable test platform. The results show that the identification accuracy reached 95.37%, and the proposed method can effectively identify the circuits of cross-bonded cables with high robustness against the domain gap, having significant engineering application value.

## 1. Introduction

High-voltage (HV) cables have gradually replaced overhead lines and become the backbone of urban power transmission networks due to their superior electrical performance and efficient use of underground space [1,2]. With the continuous increase in cable deployment, multi-circuit shared-tunnel laying has become a common practice in modern urban underground grids, enabling multiple power cable circuits to operate within a single tunnel and thereby reducing construction costs and land occupation [3,4]. Under such configurations, the accurate identification of cable circuits is one of the primary concerns for operation and maintenance personnel, as it is essential for routine inspection, fault location, maintenance planning, and emergency handling [5]. However, the high visual similarity of densely laid cables, together with aging or missing identification tags, a lack of historical records, and complex crossing arrangements, makes reliable circuit identification extremely challenging in practice. These difficulties not only degrade operation and maintenance efficiency but also increase the risk of misoperation and safety accidents. Therefore, it is necessary to investigate effective and reliable methods for cable circuit identification under multi-circuit shared-tunnel laying conditions.

Currently, circuit identification methods for overhead transmission lines mainly fall into two categories: manual inspection and signal identification. Manual inspection relies on field personnel using equipment for path verification, which is not only inefficient and costly but also imposes significant labor and safety burdens. A wireless phase checker based on plate capacitor electric field sensors was proposed in [6], which, combined with waveform transformation and zero-crossing detection, effectively realized circuit and phase sequence identification for overhead lines. A method utilizing spatial electric field distribution by installing detection probes at the top of a crane arm was proposed in [7] to identify circuits through relative electric field changes to the transmission line. Additionally, a distribution line circuit identification method based on zero-sequence current sudden changes was proposed in [8]. However, the aforementioned methods primarily target overhead lines, where mutual electromagnetic interference is minimal, and circuit paths are clear during multi-circuit operation on the same tower, making them difficult to apply directly to underground cable systems. Signal identification methods achieve circuit identification by injecting pulse currents or carrier voltages into the line and analyzing transmitted/received information. However, this approach is generally suitable prior to grid connection and involves complex operations, rendering it unsuitable for the rapid identification of cable circuits under live operation conditions.

Cross-bonding cables and their sheath grounding currents are currently hotspots of domestic and international research [9,10]. In recent years, combining sheath current characteristics with feature learning methods has emerged as a significant research direction for cross-bonding sheath topology identification [11,12]. A defect classification method for HV cable cross-bonding grounding systems based on a Logistic Regression algorithm was proposed in [13], successfully achieving the accurate identification of 27 system operating states. A classification and identification method for early cable faults based on optimized Convolutional Neural Networks (CNN) and Wavelet Transform was proposed in [14], effectively extracting overcurrent signal features to realize fault early warning. Compared to single-circuit cables, multi-circuit cables laid in shared channels exhibit complex electromagnetic coupling, generating significant induced electromotive force in the metal sheaths. A calculation model for circulating currents and current distribution in multi-circuit in-phase parallel cables was derived in [15]. On this basis, a circulating current calculation model for multi-circuit cross-bonded cable systems was further derived in [16]. The effects of mixed laying configurations and cross-bonding sheath transposition deviations on sheath currents were respectively investigated in [17,18], while the influences of laying spacing and phase sequence on sheath currents were analyzed in [19,20]. A calculation method for the electrical parameters of multi-circuit cables based on Carson’s theory and the EMTP platform was established in [21]; meanwhile, the impact of different laying methods on the symmetry of cable line parameters was analyzed in [22]. Existing research primarily focuses on suppressing sheath current elevation caused by laying asymmetry; however, accurately identifying the circuit to which a cable belongs under multi-cable shared-tunnel conditions remains a critical issue to be solved. In practice, load currents are difficult to measure directly due to trench or pipe burial [5]; simultaneously, because the segmentation positions of cross-bonded cables are consistent and certain link boxes may be installed above ground, it is impossible to distinguish circuits solely via the cable entry paths into the boxes.

In this paper, a deep embedded clustering method based on sheath current temporal characteristics was adopted to realize online circuit identification for cross-bonded cables. The main contributions of this work can be summarized as follows:

(1) A multi-circuit cross-bonded cable sheath equivalent circuit model was established, and the temporal similarity criterion for sheath currents within the same circuit was theoretically derived and validated, providing a physical foundation for online circuit identification.
(2) The robustness of the temporal similarity under various practical conditions (cable configurations, cross-bonding section length deviations, load levels, and operating modes) was systematically verified using DTW distance metrics, demonstrating the feasibility and boundary conditions of the proposed criterion.
(3) A deep embedded clustering framework combining TCN-AE and K-medoids was developed to transform circuit identification into an unsupervised temporal clustering problem. Its necessity and superiority compared to baseline methods were validated.
(4) The proposed method was validated on a full-scale 110 kV cable test platform, proving its robustness against domain shifts and demonstrating significant practical value for intelligent operation and maintenance in urban power networks.

The remainder of this paper is organized as follows. We first establish the theoretical model and identification criterion (Section 2), then verify the robustness under various conditions (Section 3). This is followed by the proposed method and simulation evaluation (Section 4) and the experimental validation on a full-scale platform (Section 5). Finally, we present our conclusions and future research directions (Section 6).

## 2. Calculation Model for Sheath Current of Multi-Circuit Shared-Tunnel HV Cables

### 2.1. Sheath Current Modeling Basis for Multi-Circuit Cross-Bonded HV Cable Systems

Circuit identification is achieved by measuring the sheath grounding currents of each cable, which consist of the phasor sum of induced current and leakage current. Theoretical analysis revealed that the three-phase sheath-current time-series within the same circuit exhibited a high degree of temporal similarity, whereas those from different circuits showed distinct temporal characteristics.

#### 2.1.1. Theoretical Background

In HV cable systems rated at 110 kV and above, single-core cables are typically employed. To ensure system safety and limit excessive induced voltage on the metallic sheath, cross-bonding grounding is commonly adopted for long cable lines. Specifically, the metallic sheath of each phase is divided into three sections of approximately equal length, and these sections are reconnected at cable joints via cross-bonding to form new sheath circuits, thereby effectively suppressing induced voltages. Meanwhile, leakage currents flow from the conductor to the metallic sheath through the main insulation and subsequently from the sheath to the ground via the outer sheath or grounding devices. Consequently, under cross-bonding configurations, the sheath circulating current originates from the combined effects of induced and leakage currents. Its temporal variation characteristics are closely related to the operating conditions, circuit layout, and spatial coupling among cables.

**Figure 1:**
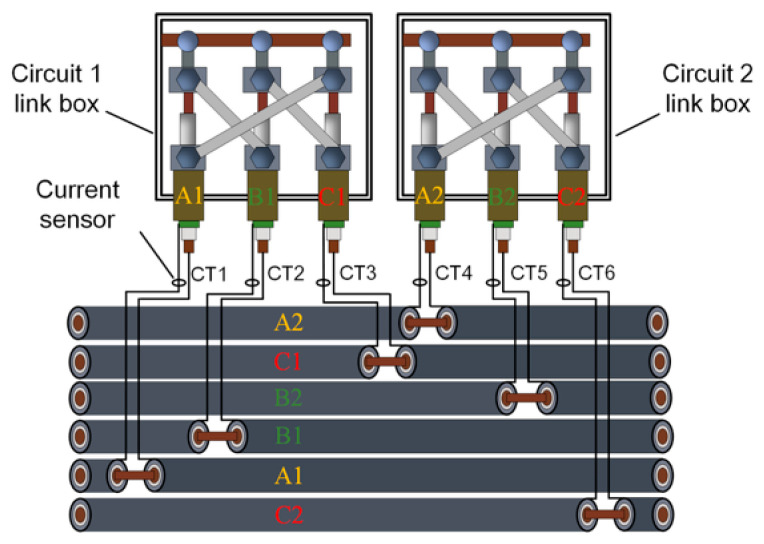
Schematic diagram of multi-circuit cable operation in a shared trench.

In multi-circuit installations where several circuits share the same trench, multiple cables are laid in parallel. Due to construction constraints and operational factors, cables from different circuits may not be arranged strictly according to the designed sequence, which can result in disordered circuit layouts and increase the difficulty of circuit identification, operation, and maintenance management. In practical engineering applications, sheath grounding is typically implemented at cable joints, and the branch leads at these joints provide convenient measurement points. This enables the direct acquisition of sheath-current signals from different cables under normal operating conditions. In this study, all subsequent analyses were based on sheath-current time-series signals measured at these joint branch leads, as illustrated in Figure 1.

In Figure 1, the link boxes for cable circuit 1 and cable circuit 2 are installed in juxtaposition, and the cables within the tunnel exhibit an interlaced layout. Furthermore, the cable bodies may become buried over long periods of operation. Consequently, due to the sheer volume of cables and the degradation of identification tags, the rapid identification of cable circuits within the tunnel is rendered difficult.

#### 2.1.2. Leakage Current

**Figure 2:**
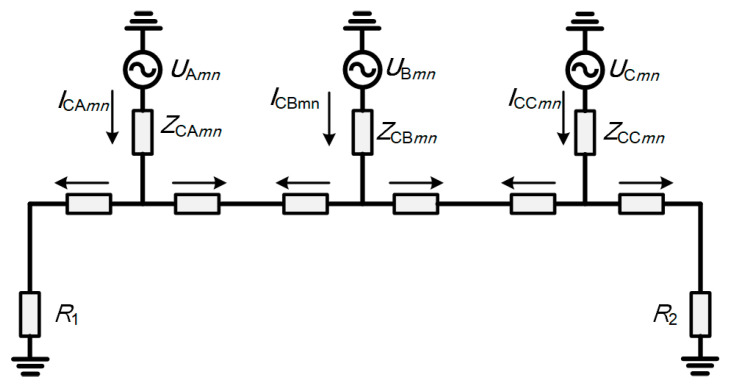
Capacitive coupling loop.

The leakage current between the conductor core and the metal sheath is shunted via impedance, contributing to the circulating current within each minor sheath section. This current comprises both capacitive and resistive components. The resistive leakage component is minimal and is typically neglected. Furthermore, since the metal sheath is directly grounded at both ends, the potential difference between the sheath and the earth is significantly smaller than that between the core and the sheath. Consequently, the leakage current flowing from the sheath to the earth is negligible and is excluded from consideration. Figure 2 illustrates the coupling circuit of the capacitive current.

The calculation of leakage current does not involve mutual coupling with other cables and is solely dependent on the intrinsic insulation characteristics of the cable itself. In Figure 2, ***U***_A*mn*_, ***U***_B*mn*_, and ***U***_C*mn*_ (*m* = 1, 2, … *p*; *n* = 1, 2, 3) are the voltage phasors in the core conductors of phases A, B, and C in the *n*th section of the *m*th circuit, in V; ***Z***_CA*mn*_, ***Z***_CB*mn*_, and ***Z***_CC*mn*_ (*m* = 1, 2, … *p*; *n* = 1, 2, 3) are the equivalent insulation impedances of the corresponding sections, which are predominantly capacitive, in Ω. The capacitive leakage current for each circuit is calculated as follows:

(1) $$\left[\begin{array}{c} I_{CAmn}\\ I_{CBmn}\\ I_{CCmn} \end{array}\right]=j\omega C\left[\begin{array}{c} U_{Amn}\\ U_{Bmn}\\ U_{Cmn} \end{array}\right]\left[{\begin{array}{ccc} l_{1} & l_{2} & l \end{array}}_{3}\right]$$

where ω is the angular frequency of the power system, *C* is the unit capacitance (capacitance per unit length) between the core and the sheath in F/m, and *l*_1_, *l*_2_, and *l*_3_ are the lengths of the three cross-bonded sections in m. The formula for calculating *C* is:

(2) $$C=\frac{2\pi \varepsilon_{r}\varepsilon_{0}}{ln((D_{c}+2\delta )/D_{c})}$$

where *ε*_r_ is the relative permittivity, *ε*_0_ is the permittivity of the vacuum, *D*_c_ is the diameter of the core, and *δ* is the thickness of the main insulation. The capacitive currents at different measurement points must be calculated individually based on impedance division.

#### 2.1.3. Induced Current

**Figure 3:**
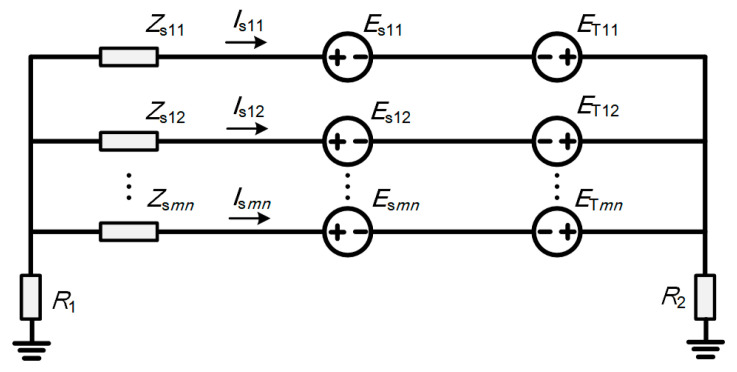
Inductive coupling loop.

In contrast to single-circuit systems, the calculation of induced voltage on metal sheaths in multi-circuit cable systems is significantly more complex. It requires a comprehensive consideration of the mutual coupling between the core currents of various circuits and the reciprocal influence of the resulting sheath circulating currents. This mutual coupling effect renders the calculation of induced current components in multi-circuit systems distinct from that in single-circuit systems, necessitating the adoption of more precise models and methods to accurately evaluate the electromagnetic characteristics of the metal sheath and their impact on the cable system. This section calculates the induced current components in multi-circuit systems based on an iterative method. Figure 3 illustrates the equivalent circuit diagram for the induced components of the sheath circulating current in multi-circuit cables.

In Figure 3, ***Z***_s*mn*_ (*m* = 1, 2, … *p*; *n* = 1, 2, 3) is the self-impedance of the *n*th sheath loop of the *m*th cable circuit, in Ω; ***I***_s*mn*_ (*m* = 1, 2, … *p*; *n* = 1, 2, 3) is the sheath circulating current in the *n*th sheath loop of the *m*th cable circuit, in A; *R*_1_ and *R*_2_ are the grounding resistances at both ends, in Ω; ***E***_T*mn*_ (*m* = 1, 2, … *p*; *n* = 1, 2, 3) is the induced voltage generated by the core currents of all circuits on the *n*th sheath loop of the *m*th circuit, in V; and ***E***_s*mn*_ (*m* = 1, 2, … *p*; *n* = 1, 2, 3) is the induced voltage generated by the sheath currents of all circuits on the *n*th sheath loop of the *m*th circuit, in V.

As the number of circuits increases, mutual coupling between more circuits must be considered, leading to an increase in the number of Kirchhoff’s Voltage Law (KVL) equations. Nevertheless, the solution methodology for each circuit remains fundamentally similar. To facilitate analysis, a double-circuit system is taken as an example below, with the calculation methods shown in Equations (3) and (4).

(3) $$\left[\begin{array}{c} E_{T11}\\ E_{T12}\\ E_{T12}\\ E_{T12}\\ E_{T12}\\ E_{T12} \end{array}\right]=\left[\begin{array}{cc} T^{11} & T^{12}\\ T^{21} & T^{22} \end{array}\right]\left[\begin{array}{c} I_{A1}\\ I_{B1}\\ I_{C1}\\ I_{A2}\\ I_{B2}\\ I_{C2} \end{array}\right]$$

(4) $$\left[\begin{array}{c} E_{s11}\\ E_{s12}\\ E_{s13}\\ E_{s21}\\ E_{s22}\\ E_{s23} \end{array}\right]=\left[\begin{array}{cc} M^{11} & M^{12}\\ M^{21} & M^{22} \end{array}\right]\left[\begin{array}{c} I_{s11}\\ I_{s12}\\ I_{s13}\\ I_{s21}\\ I_{s22}\\ I_{s23} \end{array}\right]$$

Here, ***I***_A*m*_, ***I***_B*m*_, and ***I***_C*m*_ (*m* = 1, 2) are the three-phase core currents of the *m*th circuit, in A. The complete 6 × 6 core-to-sheath coupling matrix **T** and the sheath-to-sheath coupling matrix **M** can be divided into 3 × 3 sub-blocks, explicitly distinguishing the intra-circuit coupling from the inter-circuit coupling. Let *i j* (*i*; *j* = 1, 2) denote the target and source circuit indices, yielding sub-blocks **T***^ij^* and **M***^ij^*.

Let *p q* (*p*; *q* = 1, 2, 3) represent the initial phases for the row and column of each block. The cross-bonding phase shift in the *n*th section is defined by the cyclic permutation function:

(5) $$\phi \left(p,n\right)=\left(p+n-2\right)\;mod\;3+1$$

For notational convenience, let *S* be a function mapping the numerical phase index to its corresponding letter, where *S*(1) = *A*, *S*(2) = *B*, *S*(3) = *C*. Since the core currents do not undergo cross-bonding, their phase remains static across sections. The element at the *p*th row and *q*th column of block **T***^ij^* is expressed as:

(6) $$T_{p,q}^{ij}=\sum_{n=1}^{3}X_{S{\left(\phi (p,n)\right)}_{i}S{\left(q\right)}_{j}}\cdot l_{n}$$

Under ideal equal-length cross-bonding conditions, the core-to-sheath coupling matrix **T** is neither symmetric nor circulant because the cores remain stationary while the sheaths transpose. Instead, each sub-block uniquely possesses identical rows. Mathematically, commutative scalar addition ensures equal impedance summations for each row. Physically, this indicates that the transposing sheaths uniformly share inductive fluxes from the stationary cores, resulting in induced electromotive forces of perfectly equal magnitude and phase. Conversely, since both the target and source sheath loops undergo cross-bonding simultaneously, the elements of block **M***^ij^* are expressed as:

(7) $$M_{p,q}^{ij}=\sum_{n=1}^{3}Z_{S{\left(\phi (p,n)\right)}_{i}S{\left(\phi (q,n)\right)}_{j}}\cdot l_{n}$$

The diagonal elements of this matrix are strictly zero because the sheath self-impedance is already independently accounted for on the left side of the KVL equations. Unlike matrix T, the global sheath-to-sheath coupling matrix M is inherently symmetric based on the electromagnetic reciprocity theorem. However, due to the geometric asymmetry of multi-circuit spatial arrangements, its inter-circuit sub-blocks are generally asymmetric. Furthermore, because the sheaths of each circuit undergo synchronous cyclic transpositions, **M** exhibits a strict block-circulant structure within its M sub-blocks. It is strictly not globally circulant, as a full cyclic shift would physically violate the absolute isolation between independent circuits.

*X_xiyi_* and *X_xixi_* (*x*, *y* = A, B, C; *i* = 1, 2) are the unit mutual impedance between a cable core and other cable sheaths and the unit mutual impedance and its own cable sheath, respectively, in Ω/m; *Z_xiyi_* (*x*, *y* = A, B, C; *i* = 1, 2) is the unit mutual impedance between cable sheaths, in Ω/m. The calculation formulas are shown in Equations (8)–(10).

(8) $$X_{xiyi}=2j\omega ln\frac{1}{S_{xiyi}}\times 10^{-7}$$

(9) $$X_{xixi}=2j\omega ln\frac{1}{D_{c}}\times 10^{-7}$$

(10) $$Z_{xiyi}=2j\omega ln\frac{d_{e}}{S_{xiyi}}\times 10^{-7}$$

Here, *S_xiyi_* is the center-to-center spacing between cables, in m; and *d*_e_ is the equivalent depth of earth return, in m. While leakage current usually remains at the milliampere level and is therefore negligible compared with the ampere-level induced sheath currents in electromagnetic coupling calculations, this simplification applies only to the induced-current modeling in this section, and leakage current must be retained in the measurement signal model to represent the physically measured sheath current.

(11) $$\left\{\begin{array}{c} Z_{s11}I_{s11}+\left(R_{1}+R_{2}\right)I_{s}+E_{s11}=E_{T11}\\ Z_{s12}I_{s12}+\left(R_{1}+R_{2}\right)I_{s}+E_{s12}=E_{T12}\\ Z_{s13}I_{s13}+\left(R_{1}+R_{2}\right)I_{s}+E_{s13}=E_{T13}\\ Z_{s21}I_{s21}+\left(R_{1}+R_{2}\right)I_{s}+E_{s21}=E_{T21}\\ Z_{s22}I_{s22}+\left(R_{1}+R_{2}\right)I_{s}+E_{s22}=E_{T22}\\ Z_{s23}I_{s23}+\left(R_{1}+R_{2}\right)I_{s}+E_{s23}=E_{T23} \end{array}\right.$$

By simultaneously solving Equations (3), (4), and (11), the induced currents for each sheath loop can be determined. Due to the mutual coupling between the equations, an iterative approach is required for the solution.

The calculation process is as follows. First, based on the parameter correction method in [12], the cable impedance and capacitance parameters are corrected by comparing measured sheath currents with calculated values. Subsequently, the initial induced current components are set for the three-phase sheath loops of each circuit. Since the induced voltage caused by core currents is independent of the induced currents in the sheaths, the induced current components generated solely by core currents can be used as initial values. Then, the induced currents for each circuit are calculated iteratively. Finally, the sheath current at any location can be obtained via the superposition theorem.

### 2.2. Circulating Current Analysis

The development of the multi-circuit cable coupling model has revealed the fundamental principles of current interactions in cross-bonded cable systems. However, while the model provides a framework for current coupling, it has yet to identify the specific features that can effectively distinguish between different cable circuits. To further advance cable circuit identification, it is essential to explore and extract distinguishing features.

In practical engineering applications, the sheath current measured at cable joint branch lines is a superposition of leakage and induced components; although the separation of these components can be achieved by the method in [23], which relies on synchronous sheath current measurements at grounding boxes at both ends of the cable section, such measurement configurations are overly complex for practical online implementation. Therefore, the following analysis focuses on the variation trends of the measured sheath current to investigate whether circuit-dependent similarity can be observed without explicit component separation.

When the lengths of the three cross-bonded sections are equal, i.e., *l*_1_ = *l*_2_ = *l*_3_ = *l*, the induced voltage generated by the core currents in the sheath loops of each three-phase cable circuit is expressed as:

(12) $$\begin{array}{c} E_{T11}=E_{T12}=E_{T13}=-j(M_{1A1}I_{A1}+M_{1B1}I_{B1}\\ +M_{1C1}I_{C1}+M_{1A2}I_{A2}+M_{1B2}I_{B2}+M_{1C2}I_{C2})l \end{array}$$

(13) $$\begin{array}{c} E_{T21}=E_{T22}=E_{T23}=-j(M_{2A1}I_{A1}+M_{2B1}I_{B1}\\ +M_{2C1}I_{C1}+M_{2A2}I_{A2}+M_{2B2}I_{B2}+M_{2C2}I_{C2})l \end{array}$$

where *M_ixy_* is the sum of the unit mutual impedances between the three sections of the sheath loop of the *i*th circuit (*i* = 1, 2) and the cable core of phase *x* (*x* = A, B, C) of the *y*th circuit (*y* = 1, 2). This implies that the induced voltages generated by the load currents on the three sheath loops of each cable circuit are equal. By simultaneously solving Equations (4) and (12), the relationship between the induced voltages generated by load currents and the induced currents in each sheath loop can be established through an equivalent impedance matrix **Z**_T_, as shown in Equation (14).

(14) $$\left[\begin{array}{c} E_{T11}\\ E_{T11}\\ E_{T11}\\ E_{T21}\\ E_{T21}\\ E_{T21} \end{array}\right]=\left[\begin{array}{cccccc} Z_{1} & Z_{2} & Z_{2} & Z_{5} & Z_{6} & Z_{7}\\ Z_{2} & Z_{1} & Z_{2} & Z_{6} & Z_{5} & Z_{6}\\ Z_{2} & Z_{2} & Z_{1} & Z_{7} & Z_{6} & Z_{5}\\ Z_{5} & Z_{6} & Z_{7} & Z_{3} & Z_{4} & Z_{4}\\ Z_{6} & Z_{5} & Z_{6} & Z_{4} & Z_{3} & Z_{4}\\ Z_{7} & Z_{6} & Z_{5} & Z_{4} & Z_{4} & Z_{3} \end{array}\right]\left[\begin{array}{c} I_{s11}\\ I_{s12}\\ I_{s13}\\ I_{s21}\\ I_{s22}\\ I_{s23} \end{array}\right]$$

The elements of the total equivalent impedance matrix are given by Equations (15)–(21).

(15) $$Z_{1}=R_{1}+R_{2}+Z_{s11}$$

(16) $$Z_{2}=R_{1}+R_{2}+(Z_{A1B1}+Z_{B1C1}+Z_{A1C1})l$$

(17) $$Z_{3}=R_{1}+R_{2}+Z_{s21}$$

(18) $$Z_{4}=R_{1}+R_{2}+(Z_{A2B2}+Z_{B2C2}+Z_{A2C2})l$$

(19) $$Z_{5}=R_{1}+R_{2}+(Z_{A1A2}+Z_{B1B2}+Z_{C1C2})l$$

(20) $$Z_{6}=R_{1}+R_{2}+(Z_{C1A2}+Z_{A1B2}+Z_{B1C2})l$$

(21) $$Z_{7}=R_{1}+R_{2}+(Z_{B1A2}+Z_{C1B2}+Z_{A1C2})l$$

Here, the grounding resistances and sheath loop self-impedances are absolute impedances in Ω. The mutual impedances between cable sheaths, given per unit length, must be multiplied by the corresponding section length to obtain absolute impedances, ensuring dimensional consistency of the equations.

To ensure the existence of a unique algebraic solution for the sheath currents under any cable layout, it is essential to verify the rank of the total equivalent impedance matrix **Z**_T_ presented in Equation (22). The total impedance matrix can be decomposed into a resistance matrix **R** and a reactance matrix **X**, such that:

(22) $$Z_{T}=R+jX$$

Based on Equations (15)–(21), the diagonal elements of the resistance matrix **R** are *R*_1_ + *R*_2_ + *R*_s*mn*_ (where *R*_s*ii*_ is the real part of *Z*_s*mn*_) while the off-diagonal elements are purely *R*_1_ + *R*_2_ Thus, **R** can be expressed as:

(23) $$R=(R_{1}+R_{2})J+D$$

where **J** is a 6 × 6 matrix of ones, and **D** is the diagonal matrix of the sheath self-resistances. For any non-zero real vector ***v*** = (*v*_1_, *v*_2_, *v*_3_, *v*_4_, *v*_5_, *v*_6_)^T^, the quadratic form is calculated as:

(24) $$Q=v^{T}Rv=(R_{1}+R_{2})v^{T}Jv+v^{T}Dv$$

Through calculation, we obtain:

(25) $$(R_{1}+R_{2})v^{T}Jv=(R_{1}+R_{2}){\left(v_{1}+v_{2}+v_{3}+v_{4}+v_{5}+v_{6}\right)}^{2}$$

For any non-zero phasor v, this term is always greater than or equal to 0. Furthermore, we have:

(26) $$v^{T}Dv=R_{s11}v_{1}^{2}+R_{s12}v_{2}^{2}+R_{s13}v_{3}^{2}+R_{s21}v_{4}^{2}+R_{s22}v_{5}^{2}+R_{s23}v_{6}^{2}$$

Since the sheath self-resistances *R*_s*mn*_ are inherently positive, this term is strictly greater than 0 for any non-zero phasor. Consequently, Q > 0, meaning that the matrix **R** is strictly positive definite. Since the real part R of the symmetric complex impedance matrix **Z**_T_ is strictly positive definite, **Z**_T_ is inherently non-singular (full rank). This mathematically guarantees that the governing equations possess a unique algebraic solution.

As indicated by Equation (14), the 3 × 3 sub-matrices in the top-left and bottom-right corners of the total equivalent impedance matrix exhibit a circulant symmetric structure. Therefore, under ideal conditions, the induced currents in the three sheath loops of the same cable circuit are identical.

(27) $$\left\{\begin{array}{c} I_{s11}=I_{s12}=I_{s13}\\ I_{s21}=I_{s22}=I_{s23} \end{array}\right.$$

In practical engineering, due to installation tolerances and route constraints, minor-section lengths inevitably deviate from perfect symmetry. When setting *l*_1_ = *l*(1 + δ_1_), *l*_2_ = *l*(1 + δ_2_), *l*_3_ = *l*(1 + *δ*_3_) (where *δ_i_* represents the relative deviation), the elements of the coupling matrices exhibited a strictly linear sensitivity to these deviations. Because the partial derivative of the impedance matrix element with respect to *l*_n_ is a constant unit impedance, the deviation in the matrix parameters is linearly proportional to *δ_n_*.

Although this linear sensitivity is bounded, the resulting deviations fundamentally disrupt the ideal mathematical structure of the matrices, leading to an imbalance in the induced electromotive forces and consequently to differences among the three-phase induced currents within the same circuit. Therefore, under asymmetrical configurations, relying solely on absolute current amplitude thresholds to distinguish between different circuits becomes unreliable, making it necessary to further analyze the temporal characteristics of sheath currents.

With minor modifications to the equations, this conclusion is also applicable to multi-circuit cables; that is, the induced sheath currents for the three phases within each circuit are respectively identical. Furthermore, the relationship between the load current and the induced sheath current can be derived as:

(28) $$\left[\begin{array}{c} I_{s11}\\ I_{s12}\\ I_{s13}\\ I_{s21}\\ I_{s22}\\ I_{s23} \end{array}\right]=P\left[\begin{array}{c} I_{A1}\\ I_{B1}\\ I_{C1}\\ I_{A2}\\ I_{B2}\\ I_{C2} \end{array}\right]$$

The matrix **P** is defined as the transfer coefficient matrix between the core current and the induced sheath current, with its elements derived from the simultaneous Equations (3), (4), and (11). Once the cable layout and positioning are determined, the mutual inductance between any two phases of the cable is fixed, and the elements of the matrix no longer change. Therefore, the matrix **P** becomes a constant matrix.

The measured sheath current is the phasor sum of the induced current and the leakage current. The leakage current is primarily influenced by insulation parameters and remains unaffected by load fluctuations. In contrast, the induced current is directly controlled by the core current through the matrix **P**, and it exhibits dynamic fluctuations that are consistent with the load current. Theoretically, if the constant leakage current component can be isolated and only the induced current, which reflects the time-varying characteristics of the load, is retained, the accuracy of circuit identification would be significantly enhanced. While the method in [23] provides a way to separate these components, it requires synchronous measurement of the sheath current at the grounding boxes at both ends of the cable section, making it a relatively complex setup for practical online applications. Therefore, this paper seeks to explore an alternative approach that eliminates the need for physical separation of the current components.

### 2.3. Definition of Identification Criterion Based on Temporal Similarity

Based on the coupled circuit model derived in Section 2.1 and Section 2.2, the sheath current ***I***_CT*k*_ (*k* = 1~6) measured at the link box is a superposition of the leakage component ***I***_leak_ (which represents the phasor sum of the leakage currents flowing through the measurement point from the respective cross-bonding minor sections) and the induced component ***I***_s*mn*_. To establish a mathematical criterion for circuit identification, the current at the measurement points shown in Figure 1 can be expressed as:

(29) $$I_{CTk}(t)=I_{leak}+I_{smn}(t)+\varepsilon (t)=I_{leak}+\sum_{k=A,B,C}\alpha_{k}I_{km}+\varepsilon (t)$$

where ***I***_leak_ represents the slowly varying leakage current, α_k_ is the transfer coefficient derived from matrix **P** in Equation (28), and ε denotes measurement noise.

Since the leakage component ***I***_leak_ is fundamentally determined by insulation impedance and remains independent of load fluctuations, the dynamic fluctuation feature ***I***_CT*k*_ is primarily governed by the load current ***I****_k_*_m_ of the corresponding circuit. For any two cable channels *i* and *j*, their circuit membership relationship can be determined by the correlation of their temporal fluctuations. We defined the Temporal Fluctuation Similarity S(*i*, *j*) as a normalized metric mapped from the distance between the measured currents ***I***_CT*k*_. Based on this physical coupling mechanism, the identification criterion was established as the distinct separability between intra-circuit and inter-circuit similarities:

(30) $$\left\{\begin{array}{c} S(i,j)\geq S_{1},Circuit(i)=Circuit(j)\\ S(i,j)\leq S_{2},Circuit(i)\neq Circuit(j) \end{array}\right.$$

Ideally, sheath-current signals from cables belonging to the same circuit should exhibit a structural similarity close to 1.0. In practice, however, measurement noise, load fluctuations, and complex operating conditions cause the observed waveforms to deviate from this ideal behavior. According to established statistical conventions, correlation coefficients exceeding 0.7 are generally regarded as indicating strong similarity. Therefore, a similarity coefficient of 0.7 was adopted in this study as a robust lower bound for “same-circuit” identification; this threshold is also empirically validated in Section 3. Based on this criterion, Table 1 summarizes the quantitative criteria for separating intra-circuit *S*_1_ and inter-circuit *S*_2_. Nevertheless, under low signal-to-noise ratio conditions, similarity values may fall into an intermediate range where intra- and inter-circuit distributions partially overlap. In this regime, simple threshold-based decision rules become unreliable. This observation motivates the adoption of the deep embedded clustering method in Section 4 to learn more discriminative representations for robust circuit identification.

## 3. Similarity Analysis of Sheath Currents

The previous analysis indicates that although the sheath current is a superposition of an approximately constant leakage component and a load-driven induced component, the induced component may still retain circuit-discriminative fluctuation patterns. In practice, however, the observable temporal similarity among sheath-current signals can be affected by multiple factors: (i) cable arrangement, spacing, and phase sequence in a shared tunnel, which modify mutual coupling and the effective transfer relationship between conductor currents and sheath currents; (ii) cross-bonding minor-section length deviation, which breaks the ideal symmetry of sheath loops and alters current distribution; (iii) load level and fluctuation amplitude, which directly determine the signal-to-noise ratio (SNR) of the induced component relative to noise and leakage; (iv) operating modes, especially when two circuits experience highly correlated load fluctuations, which can increase inter-circuit similarity; and (v) measurement imperfections such as sensor noise, bias, and synchronization jitter, which introduce local distortions and time misalignment. This section therefore aims to, taking a double-circuit system as an example, quantitatively verify whether intra-circuit similarity remains consistently higher than inter-circuit similarity under the above perturbations, thereby validating the proposed “fluctuation similarity” criterion and motivating the need for more robust representation learning in Section 4.

### 3.1. DTW-Based Similarity Metric and Evaluation Protocol

A straightforward point-to-point metric (e.g., Euclidean distance or Pearson correlation) implicitly assumes strict temporal alignment between two sequences. This assumption is often violated in multi-channel sheath-current acquisition and preprocessing. For example, slight sampling asynchrony across channels, filtering/denoising phase lag, and coupling-induced differences in transient response can lead to local time shifts or non-linear temporal misalignment even when two signals share the same underlying fluctuation pattern. DTW was therefore adopted because it measures morphological similarity under flexible alignment, allowing for a more faithful comparison of fluctuation patterns when local misalignment exists. In this work, DTW was used as an analytical tool to test the robustness of the temporal similarity criterion across scenarios, rather than as the final online identification method.

Let ***x*** = (*x*_1_, …, *x*_n_) and ***y*** = (*y*_1_, …, *y*_n_) denote two preprocessed sheath-current sequences. DTW computes the minimum cumulative alignment cost through dynamic programming:

(31) $$\gamma (i,j)=d(x_{i},y_{i})+min\{\gamma (i-1,j),\gamma (i,j-1),\gamma (i-1,j-1)\}$$

where d(*x*_i_, *y*_i_) is the point-wise cost (*x_i_*−*y_i_*)^2^. The DTW distance is then obtained as γ(*i*, *j*). To make scores comparable across alignments, we used a length-normalized DTW distance.

(32) $$D_{DTW}(x,y)=\frac{\gamma (N,M)}{L}$$

where L is the length of the optimal warping path. Since the paper reports similarity in a bounded [0, 1] form for intuitive comparison, the DTW distance is mapped to a similarity coefficient:

(33) $$S(x,y)=exp\left(-\frac{D_{DTW}\left(x,y\right)}{\sigma }\right)\in [0,1]$$

where σ is a scale parameter controlling the decay rate. In implementation, σ can be set using a reference scenario, ensuring stable and reproducible similarity scores across experiments. This monotonic mapping preserves DTW’s ordering such that a smaller distance indicates higher similarity while providing an interpretable similarity coefficient for heatmap visualization and scenario comparison. For each simulated scenario, we computed pairwise DTW similarity S among all measured sheath-current channels, and organized the results into a similarity matrix visualized as a heatmap, where a higher value indicates higher similarity. The key comparison principle is consistent across all subsections: intra-circuit similarity among pairs belonging to the same circuit should remain statistically higher than inter-circuit similarity between pairs from different circuits.

### 3.2. Analysis of Influencing Factors

#### 3.2.1. Impact of Configuration and Cross-Bonding Section Length on Sheath Current Similarity

**Figure 4:**
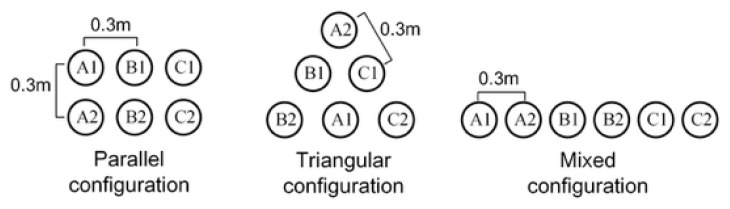
Schematic of different configurations.

To examine the robustness of the similarity criterion against geometric and coupling variations, a double-circuit cross-bonded HV cable system was constructed in PSCAD/EMTDC. PSCAD/EMTDC is suitable for modeling transient electromagnetic interactions in cable systems, enabling controlled experiments under different configurations and operating conditions. The key simulation parameters are summarized in Table 2. Specifically, the geometric parameters investigated include a fixed cable phase spacing of 0.3 m, while the laying configurations comprise parallel, triangular, and mixed arrangements, as illustrated in Figure 4. By adjusting the load, the core current was varied within the range of 0–1000 A to represent different operating conditions.

In addition, to evaluate the impact of cross-bonding length deviations, the middle section length was kept constant at 500 m, whereas the lengths of the two end sections were set with deviations of 0%, 10%, and 20%, respectively. Gaussian noise was added to the simulated sheath-current measurements to emulate practical acquisition noise. Prior to similarity evaluation, all sequences were then preprocessed using denoising and normalization procedures to reflect the signal-conditioning steps typically applied in practical monitoring systems. After preprocessing, DTW-based similarity coefficients were computed for all channel pairs and visualized as heatmaps.

**Figure 5:**
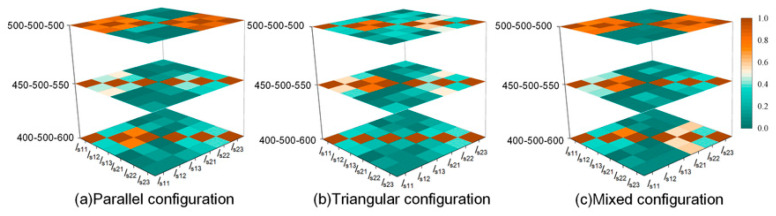
Heatmap of time-series correlation under different cable configurations.

The results in Figure 5 show that during double-circuit operation, sheath-current fluctuations from different circuits exhibited non-zero similarity due to electromagnetic coupling between circuits. Nevertheless, intra-circuit similarity remained consistently higher than inter-circuit similarity across different arrangement patterns. When the minor-section lengths in the cross-bonding scheme were identical, the DTW similarity coefficients among the three cables within the same circuit concentrated in the high range of 0.71~0.83, indicating strong temporal consistency in load-driven fluctuation patterns. In contrast, the similarity coefficients between different circuits were distributed within the lower interval of 0.22~0.61.

When cross-bonding minor-section length deviation increased, intra-circuit similarity decreased slightly, which is expected because uneven section lengths alter the effective sheath-loop impedance structure and redistribute induced currents. Across all configurations, intra-circuit similarity remained higher than inter-circuit similarity, demonstrating that the proposed fluctuation-similarity criterion is robust to common geometric and coupling variations in shared-tunnel multi-circuit laying.

#### 3.2.2. Impact of Load Level on Sheath Current Similarity

The induced component of sheath current is driven by conductor-current fluctuations; therefore, the amplitude of load fluctuation directly affects the magnitude of the informative (load-driven) component in the measured signal. Under low-load operation, sheath-current amplitude decreases, and the relative contribution of noise and approximately constant leakage components increases, effectively reducing SNR. This can weaken discriminability because similarity becomes more sensitive to noise-induced distortion than to genuine load-driven fluctuation patterns.

**Figure 6:**
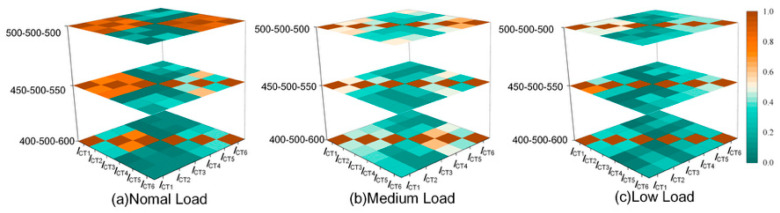
Heatmap of time-series correlation under different load levels.

To evaluate this effect, sheath currents were simulated under multiple load-fluctuation ranges and assessed using DTW similarity, as shown in Figure 6. Under normal load conditions, intra-circuit similarity coefficients remained high, ranging from 0.7~0.8. As the load level decreased to medium-to-low conditions, intra-circuit similarity reduced to the interval of 0.52~0.66, reflecting SNR degradation. Under extremely low load conditions, intra-circuit similarity could decline further to levels between 0.28 and 0.42, implying that the similarity distribution may increasingly overlap with inter-circuit similarity in this worst-case SNR regime. These observations confirm that load level is a critical factor affecting similarity-based discrimination, and they motivate the need for stronger feature extraction and longer acquisition windows to reliably capture weak fluctuation patterns under low SNR.

#### 3.2.3. Impact of Operating Modes on Sheath Current Similarity

**Figure 7:**
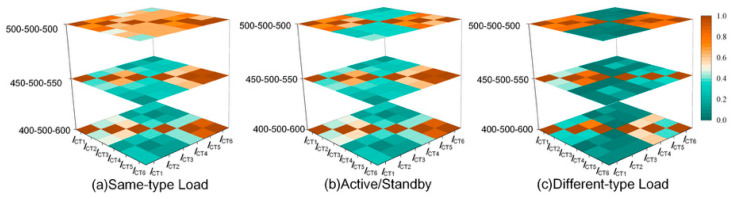
Heatmap of time-series correlation under different operating conditions.

Considering that certain double-circuit cable systems utilize specific operating modes, similarity measures were performed on sheath currents under these special conditions, with the results illustrated in Figure 7. These modes were categorized into three types: when both circuits operated with the same-type load, the load currents of the two cables exhibited highly similar fluctuations; in the active/standby mode, one circuit was operational while the other remained in a warm standby state; and when the circuits operated with different-type loads, the load currents of the two circuits typically differed significantly.

The results indicate that, under the primary-standby mode and the different-load operating mode, the similarity coefficients of sheath currents within the same circuit were generally maintained in the range of 0.60~0.85, whereas those between different circuits mostly fell within 0.25~0.52. This clear separation between intra-circuit and inter-circuit similarities indicates that the proposed criterion remains stable under these operating conditions.

In contrast, under the same-load operating mode, conductor-current fluctuations in the two circuits become highly comparable, which significantly increases the similarity of sheath currents between different circuits. As a result, inter-circuit similarity may rise to approximately 0.55~0.75, narrowing the separation from intra-circuit similarity and potentially degrading circuit identification performance. This operating condition, therefore, imposes higher robustness requirements, making it necessary to extend the acquisition duration and adopt more discriminative feature extraction and clustering methods to reduce the risk of misclassification.

The above analyses confirm that intra-circuit sheath-current similarity is generally higher than inter-circuit similarity, supporting temporal fluctuation similarity as a feasible criterion for circuit discrimination. At the same time, the results also revealed two practically important boundary conditions: (i) extremely low-load operation, where SNR is poor and similarity becomes sensitive to noise and leakage dominance; and (ii) same-load operating mode, where inter-circuit similarity rises because both circuits are driven by highly correlated load fluctuations. Under these boundary conditions, the similarity distributions of different circuits may partially overlap, making purely DTW-based or rule-based thresholding unreliable for online identification. This partial overlap highlights a critical limitation: purely relying on raw DTW-based hard thresholds is insufficient for reliable identification under severe boundary conditions. This inherent limitation directly necessitates the deep embedded clustering approach (TCN-AE) proposed in Section 4, which projects these raw overlapping sequences into a more robust latent feature space to ensure high-accuracy identification.

## 4. Circuit Identification Method Based on Deep Embedded Clustering

The previous section established the temporal similarity criterion: sheath-current time-series from the same circuit tended to exhibit higher fluctuation similarity than those from different circuits. DTW was adopted there as a robust similarity metric to validate this criterion under flexible alignment. However, under non-ideal conditions (e.g., unequal minor-section lengths, low-load SNR degradation, and highly correlated operating modes), the separation between intra-circuit and inter-circuit similarity can diminish, making threshold-based DTW decision rules or manual interpretation unreliable and difficult to generalize. Moreover, directly applying DTW online requires repeated pairwise comparisons and scenario-dependent thresholds, which are computationally and operationally undesirable. Therefore, this study formulated circuit identification as an unsupervised clustering problem on sheath-current time-series data, and introduced a deep embedded clustering framework to operationalize the criterion with improved robustness to noise and modeling errors.

### 4.1. Problem Formulation and Input–Output Definition

Let X∈R^C×T^ denote a synchronous measurement window of sheath currents, where C is the number of measured channels, and T is the number of time samples in the window. To evaluate the model’s scalability beyond the two-circuit theoretical derivation in Section 2, a four-circuit system was employed here. For a four-circuit system, C = 12 (three phases per circuit). The goal is to output a circuit membership assignment for each channel:

(34) $$\hat{y}=\{\hat{y_{1}},\ldots \hat{y_{C}}\},\hat{y_{C}}\in \{1,\ldots ,K\}$$

The proposed online identification pipeline follows an input–algorithm–output structure: the input is a multi-channel synchronous sheath-current window X; each channel is first preprocessed, then mapped into a low-dimensional feature space to obtain noise-robust temporal representations, and finally the representations are grouped via unsupervised clustering using a similarity measure consistent with the temporal similarity criterion; the output is the channel grouping ŷ for online circuit identification.

### 4.2. Deep Embedded Clustering Framework

#### 4.2.1. Temporal Convolutional Network

A Temporal Convolutional Network (TCN) is a convolutional structure designed for sequence modeling [24]. By combining one-dimensional CNNs with causal convolution, dilated convolution, and residual connections, TCN can model long-range temporal dependencies while maintaining parallel computation efficiency. Compared with recurrent structures, TCN offers stable training and strong feature extraction for time-series signals. Unlike RNNs/LSTMs, which process data sequentially, TCN’s dilated convolutions enable a large receptive field to capture long-term load fluctuation trends, while its convolutional nature captures local transient features. This matches the complex temporal dynamics of sheath currents.

#### 4.2.2. Autoencoder

An autoencoder (AE) learns representations by reconstructing input data in an unsupervised manner [25]. The encoder maps the input into a latent representation, and the decoder reconstructs the input from the latent features. Model parameters are trained to minimize the reconstruction error.

In this work, the AE was used primarily as an unsupervised representation learner to suppress nuisance components (e.g., quasi-constant leakage bias, measurement noise, and residual temporal distortions) while preserving load-driven fluctuation patterns relevant to circuit discrimination. This improves clustering stability when raw-signal similarity separation is weakened under challenging operating conditions.

#### 4.2.3. Network Design

**Figure 8:**
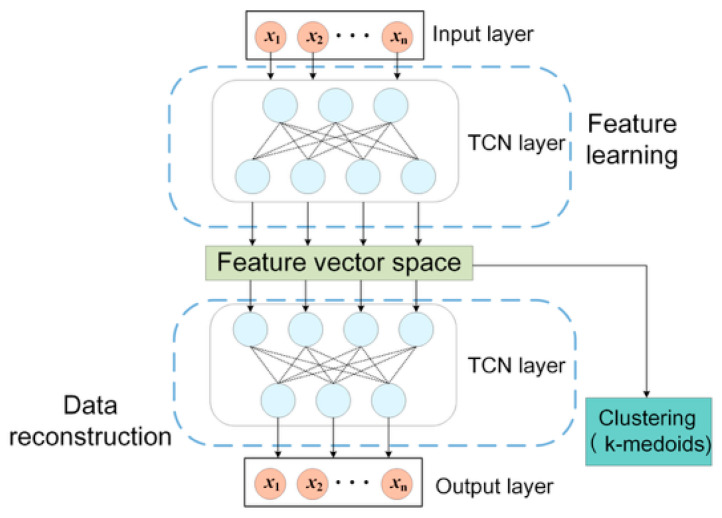
Network framework.

This paper constructed a TCN-AE to extract sheath-current time-series features, as illustrated in Figure 8. The encoder replaces fully connected layers with TCN blocks to extract local and multi-scale temporal features. The decoder reconstructs the input via convolution to enforce informative latent features.

After training, K-medoids clustering was applied to the extracted feature vectors to determine circuit membership. Compared with K-means, K-medoids selects actual samples as cluster centers, improving robustness to outliers and noise.

### 4.3. Case Study Analysis

To quantitatively analyze the feasibility of the proposed model in cable circuit identification and to measure the impact of cross-bonding grounding system parameters on clustering results, this paper extends the sheath current calculation model from Section 2 to a four-circuit system. This generates sheath current time-series data containing various operating conditions for model training. The dataset comprises a total of 900 samples, with each sample containing 12 cable sheath-current time-series, resulting in a total of 10,800 time series. The dataset was divided into a training set and a testing set in a 4:1 ratio. Based on this, the features of multi-circuit sheath currents were analyzed and clustered to effectively evaluate the applicability and accuracy of the identification algorithm in practical engineering.

**Figure 9:**
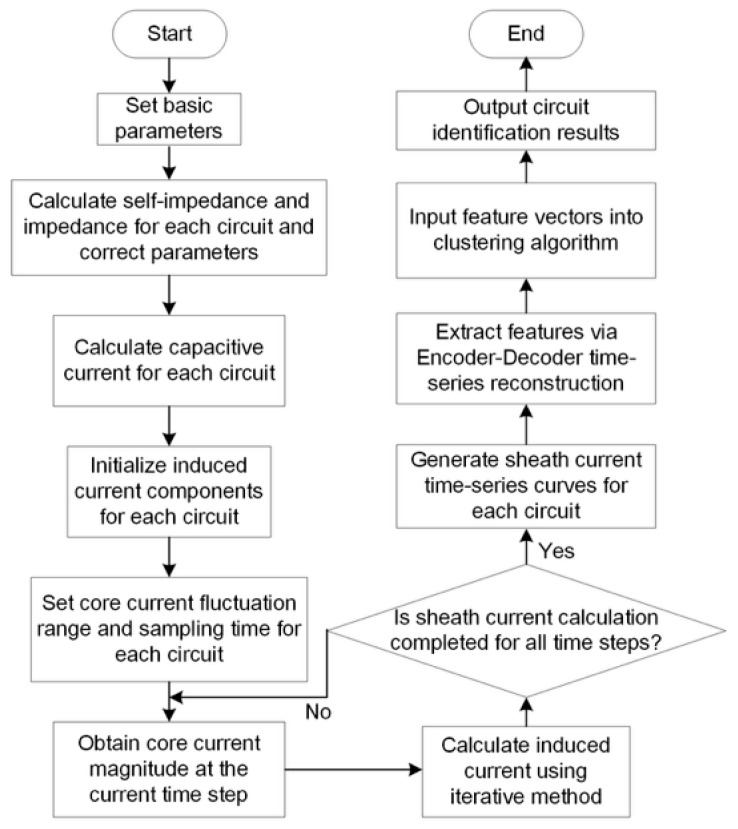
Flowchart of circuit identification.

Considering the complexity of the cable operating environment and the diversity of laying methods, the calculation of sheath currents involved randomly setting parameter variations for cable distance *S*, phase spacing *l*, and load current *I*, as shown in Equation (35). Furthermore, slight stochastic perturbations were introduced to the cable’s intrinsic structural parameters to account for manufacturing tolerances and measurement uncertainties. This domain randomization strategy ensured that the generated dataset encompassed the stochastic variability of real-world environments, thereby maximizing the model’s robustness and ability to generalize from simulation to actual field conditions. Figure 9 summarizes the overall pipeline. Given a time window of 12-channel synchronous sheath-current measurements, the signals are preprocessed and fed into a TCN-AE to obtain low-dimensional temporal representations. These representations are then clustered by K-medoids to infer circuit membership (i.e., grouping the three phases belonging to the same circuit). For quantitative evaluation, cluster indices are aligned with ground-truth circuit labels using a post hoc label-matching procedure.

(35) $$\left\{\begin{array}{c} S=\left[0.2\;m,0.5\;m\right]\\ l=\left[400\;m,500\;m\right]\\ I=\left[100\;A,1000\;A\right] \end{array}\right.$$

First, the preprocessed sheath-current time-series data are normalized via the input layer. It then passes through three TCN encoder blocks, where each block consists of two dilated causal convolution layers (with a kernel size of 3 and dilation factors of 1, 2, and 4, respectively), a normalization layer, a ReLU activation layer, and a Dropout layer (rate set to 0.1), incorporating residual connections. Subsequently, the data pass through a feature extraction layer followed by three TCN decoder blocks, which share the same structure as the encoder. The entire model adopts a two-stage training strategy. The first step is called the TCN-AE pre-training stage. The Adam optimization algorithm was used with an initial learning rate of 0.001. A piecewise learning rate adjustment strategy was employed, reducing the learning rate by 50% every 50 epochs. The maximum number of training epochs was set to 200, the gradient threshold to 1.0, and the L2 regularization coefficient to 1×10^−4^. The TCN encoder–decoder parameters were pre-trained by minimizing the reconstruction error to learn the low-dimensional feature representation of the data. In the subsequent phase, the feature extraction and K-medoids clustering optimization, the pre-trained TCN encoder was used to extract feature vectors, and the K-medoids clustering algorithm was applied for unsupervised classification. The number of clusters corresponded to the number of cable circuits. The DTW distance was used as the metric, with a maximum of 100 iterations. Clustering was performed in groups of 12 samples, and a correction function mapped the cluster labels to the true category labels, achieving an effective combination of deep feature learning and unsupervised clustering.

### 4.4. Evaluation and Comparison

#### 4.4.1. Metrics and Label Alignment for Clustering Evaluation

**Figure 10:**
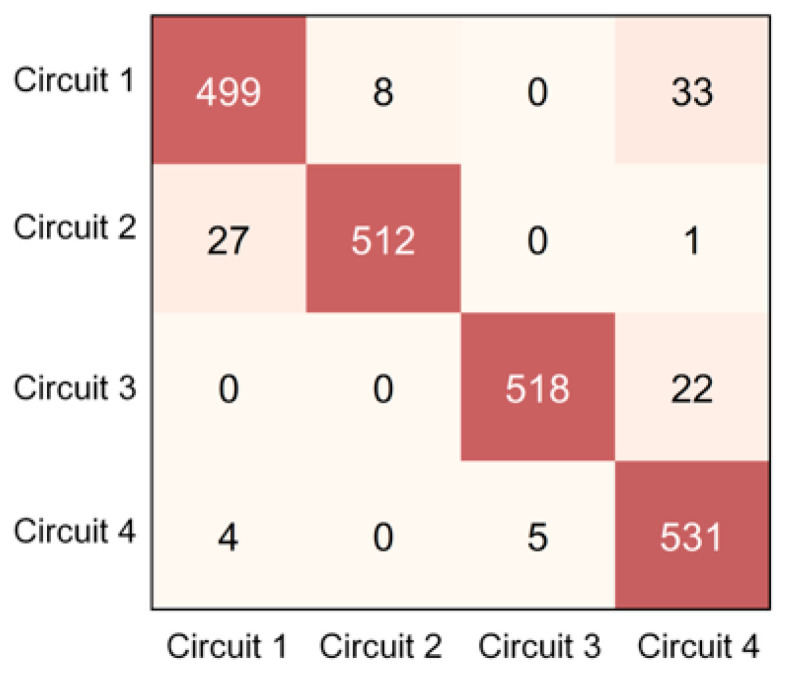
Confusion matrix of recognition results.

Although circuit identification is fundamentally an unsupervised clustering task, classification-style evaluation metrics are used to quantify its reliability in real-world engineering scenarios. To resolve the correspondence between clustering results and actual labels, a post-processing exhaustive permutation method was employed. By iterating through all possible label mappings, this method identifies the optimal alignment between predicted clusters and ground-truth circuits, ensuring that the reported Accuracy, Precision, Recall, and F1-score accurately reflect the model’s identification performance, as shown in Figure 10. In the confusion matrix, columns represent predicted circuit labels and rows represent ground-truth circuit labels.

Furthermore, to rigorously assess the intrinsic geometric quality and label consistency of the learned representations without relying exclusively on classification mappings, a complementary set of clustering-oriented evaluation criteria was employed. We utilized the Adjusted Rand Index (ARI) and Normalized Mutual Information (NMI) to measure the agreement between the clustering results and the ground-truth partitions up to permutation. Additionally, the Davies–Bouldin Index (DBI) and the Silhouette Coefficient were evaluated to reflect the intra-cluster compactness and inter-cluster separation, where a lower DBI and a higher Silhouette score indicate superior topological structure.

As presented in Table 3, the model achieved an ARI of 0.581 and an NMI of 0.665, indicating a robust correlation with the underlying semantic grouping of the physical circuits. Concurrently, the structural metrics (DBI = 0.303, Silhouette = 0.865) demonstrated that the latent space representations formed highly cohesive and strictly separated cluster boundaries. These unsupervised metrics, combined with the aligned classification scores and the confusion matrix evaluated in the subsequent section, comprehensively validate the discriminative power of the proposed method.

#### 4.4.2. Quantitative Results and Ablation Study

An ablation study was conducted to validate the effectiveness of the proposed framework. Table 4 compares four models: (i) TCN-AE + K-medoids, (ii) TCN-AE + K-means, (iii) LSTM-AE + K-medoids, and (iv) K-medoids without deep feature learning. The proposed method achieved the best overall performance. In the testing set (180 samples, corresponding to 2160 time series in total and 540 time series per circuit), the evaluation metrics of the proposed model (Accuracy, Precision, Recall, and F1-score) all exceeded 95%, indicating superior circuit identification capability compared with the baseline alternatives.

**Figure 11:**
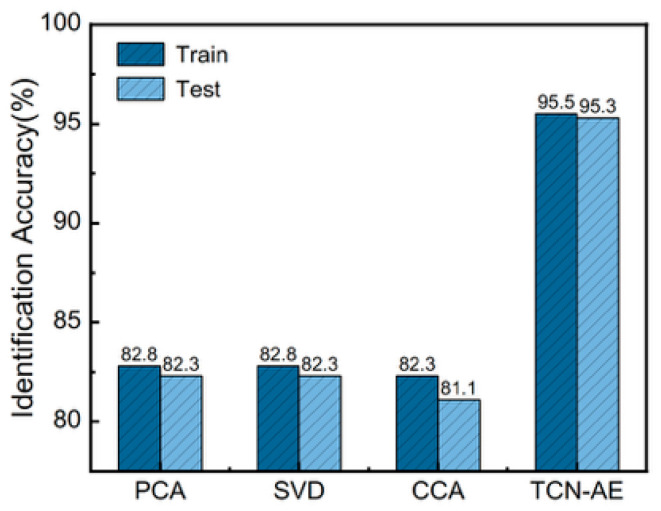
Performance comparison of different baseline models.

To rigorously validate the necessity of the proposed nonlinear deep feature extraction, several linear dimensionality reduction techniques—specifically, Principal Component Analysis (PCA), Singular Value Decomposition (SVD), and Canonical Correlation Analysis (CCA)—were implemented as comparative baselines. The latent features extracted by each respective method were subsequently clustered using the K-medoids algorithm. To ensure a strictly fair comparison, the final identification accuracy for all methods was evaluated on the exact same train/test data split., see Figure 11.

As demonstrated, the proposed nonlinear TCN representation significantly outperformed all linear baselines, achieving at least a 12% accuracy improvement over PCA. Notably, the failure of label-assisted CCA to surpass unsupervised PCA confirms that the primary bottleneck is the inherent inability of linear projections to capture complex temporal dynamics, rather than a lack of label supervision. Physically, sheath-current waveforms frequently exhibit local time shifts and multi-scale fluctuations. Linear methods relying on fixed temporal alignment and global variance severely struggle with these dynamic variations. In contrast, the proposed TCN leverages dilated causal convolutions to effectively aggregate multi-scale temporal information, successfully preserving the critical fluctuation patterns necessary for robust circuit discrimination under diverse operating conditions.

#### 4.4.3. Latent-Space Visualization

**Figure 12:**
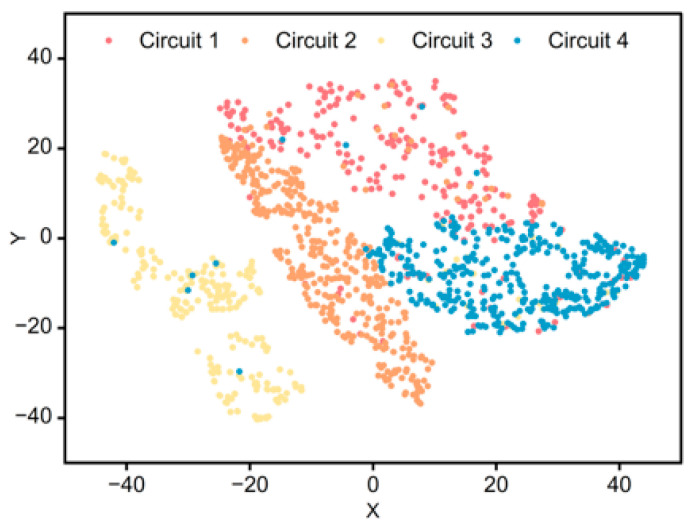
Feature distribution visualization via t-SNE.

To visually validate the discriminative capability of the learned representations, the t-SNE (t-Distributed Stochastic Neighbor Embedding) algorithm was applied to project the high-dimensional feature vectors from the test set into a 2D space. Figure 12 displays the resulting feature distribution. It can be observed that the samples from the four different circuits form four distinct and compact clusters with clear boundaries. Despite the potential similarities in raw waveforms mentioned in Section 3, the TCN-AE network successfully mapped them into separable regions in the latent feature space. This high degree of separation explains the model’s superior clustering accuracy and validates its robustness in extracting circuit-specific temporal patterns.

#### 4.4.4. Representation Diagnostics and Latent Space Analysis

To rigorously evaluate the learned representation beyond qualitative visualizations, we introduced two quantitative assessments. First, we compared the intra- and inter-circuit distances across both the original signal space and the latent feature space to verify whether the encoding process genuinely amplified circuit separability. Second, we tracked the correlation between reconstruction fidelity and clustering quality, establishing a direct link between representation learning and practical identification performance. To confirm that the latent mapping significantly enhanced the physical similarity structure identified in Section 3, we comprehensively evaluated pairwise distances across four distinct measurement settings by applying both Euclidean and DTW metrics to the raw signals (as baselines) and to the learned latent features. This cross-comparison robustly quantified the representational gain. To formalize this analysis, the dispersion ratio *r* was mathematically defined as follows:

(36) $$r=\frac{{\bar{d}}_{\mathrm{intra}}}{{\bar{d}}_{\mathrm{inter}}}$$

(37) $${\bar{d}}_{\mathrm{intra}}=\frac{1}{\left|P_{\mathrm{intra}}\right|}\sum_{(i,j)\in P_{\mathrm{intra}}}d(i,j)$$

(38) $${\bar{d}}_{\mathrm{inter}}=\frac{1}{\left|P_{\mathrm{inter}}\right|}\sum_{(i,j)\in P_{\mathrm{inter}}}d(i,j)$$

Here, *P*_intra_ and *P*_inter_ denote the sets of channel pairs belonging to the same circuit and to different circuits, respectively. The term d(*i*, *j*) represents the pairwise distance between channels *i* and *j*, computed according to the metric specific to the evaluated space. A smaller dispersion ratio r indicates that intra-cluster distances are significantly tighter than inter-cluster distances, reflecting a more compact and strictly separated latent structure. To explicitly quantify the representational improvement achieved by the encoding process, we defined the relative reduction in Δ*r* as follows:

(39) $$\Delta r=\frac{r_{\mathrm{raw}}-r_{\mathrm{latent}}}{r_{\mathrm{raw}}}\times 100\%$$

**Figure 13:**
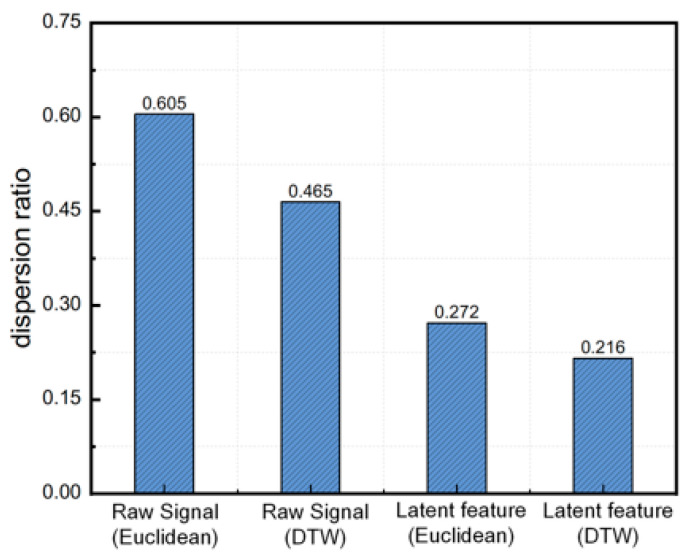
Separability comparison.

Initially, the dispersion ratios in the raw space were all below 0.8, indicating an inherent degree of separability that aligned with the preceding analysis. As illustrated in Figure 13, the dispersion ratio r exhibited a substantial decrease after deep representation learning across different distance metrics. Under the Euclidean metric, *r* dropped from 0.605 in the raw space to 0.272 in the latent space, achieving a reduction of 55.0%. More importantly, when evaluated with the DTW metric consistent with the final K-medoids clustering configuration, the ratio was further minimized from 0.465 to an optimal 0.216, representing a 53.5% decrease. These results confirm that the TCN-AE encoded features effectively preserve intra-circuit similarity patterns while maximizing the distance margin between different circuits, fully validating the robustness of the learned latent representation in offline analysis.

To formally establish the causal relationship between the autoencoder’s unsupervised reconstruction task and the ultimate clustering success, we tracked the evolution of the latent feature space in the TCN-AE’s training progression. We systematically extracted network snapshots at these specific intervals and evaluated two metrics for each stage: the Reconstruction Loss (MSE) and the corresponding Silhouette Score of the K-medoids clustering.

**Figure 14:**
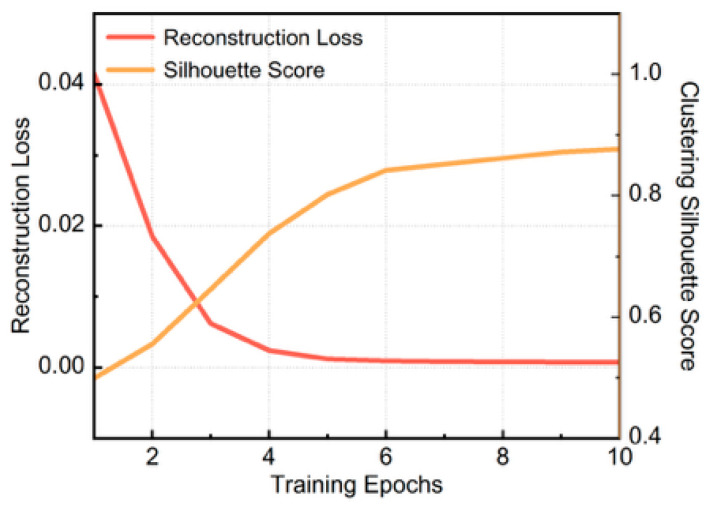
Correlation between reconstruction fidelity and clustering quality.

As illustrated in Figure 14, there was a strong inverse correlation between these two metrics. Before the model converged, the network exhibited a higher reconstruction error, indicating that it had not yet learned to accurately restore the multi-circuit sheath currents. Consequently, the extracted latent features mapped to a highly overlapping and less separable manifold, resulting in a lower Silhouette score. As the TCN-AE was forcefully constrained by the MSE objective to filter out nuisance variables and learn the intrinsic temporal structure, the reconstruction loss rapidly dropped to a stable low value. Correspondingly, the Silhouette score significantly surged and stabilized near 0.85. This correlated trajectory definitively proves that the robust geometric separability of the cable circuits is not trivially inherent to the raw data, but is progressively constructed and strictly sustained by the autoencoder’s representation learning fidelity.

## 5. Field Application and Case Analysis

To evaluate the engineering applicability of the proposed similarity-based identification criterion, the method was implemented on a full-scale cable test platform. The experiment examined whether the temporal similarity of sheath currents could be reliably maintained under complex operating conditions, including multi-circuit electromagnetic coupling and dynamic load fluctuations. The results demonstrate that the high-similarity characteristics of sheath currents within the same circuit were effectively preserved in the test environment.

### 5.1. Test Platform and Measurement Setup

In real cross-bonded HV cable systems, circuit identification is challenging because cable routes are visually indistinguishable in shared tunnels, and load currents are often unavailable for direct measurement. This work enables online circuit identification using only synchronously measured sheath-current time-series at link-box branch leads, without signal injection or topology interruption. To obtain stable decisions under noise and short-term ambiguity, the pretrained TCN-AE representation and K-medoids clustering are deployed on-site together with a sliding-window stability criterion.

**Figure 15:**
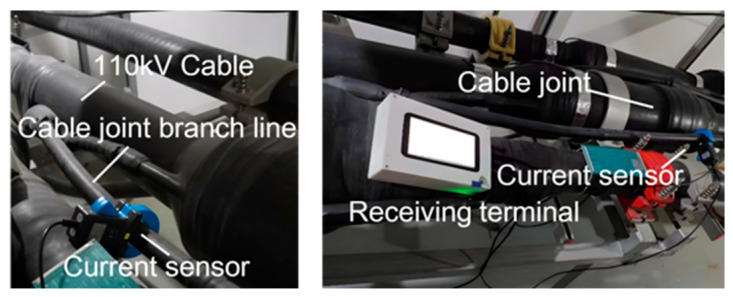
Field test diagram.

As illustrated in Figure 15, current sensors were installed at the cross-bonding link branch lines of each cable, with the number of sensors matching the number of cables to ensure that each sheath current was acquired independently and synchronously. The sensors utilized were ferromagnetic current transformers with a transformation ratio of 200/5. During the test, the actual sheath currents ranged from 20 to 60 A; this operating range was within the linear region of the current sensors, ensuring measurement accuracy. The data acquisition system was configured with an appropriate input range to optimize the signal resolution and signal-to-noise ratio for the observed current levels. The sensors were connected to the acquisition channels via shielded cables, and the collector communicated with the receiving terminal through a USB interface.

### 5.2. Online Deployment Pipeline and Stability Strategy

The receiving terminal embeds the pretrained clustering model. Raw sheath-current measurements are first preprocessed and then fed into the TCN-AE encoder to obtain low-dimensional temporal representations, which are clustered by K-medoids to infer circuit membership by grouping the three phases belonging to the same circuit. The software interface outputs the channel indices of the three cables assigned to each circuit and stores the corresponding waveforms and identification results after acquisition.

To prevent label instability caused by short-term fluctuations and noise, a time-window stability criterion was employed for online decision making. Sheath-current data were processed in a sliding window of length *W* with hop size H. Let *π*_t_ denote the channel-to-circuit grouping result obtained from the *t*th window, subject to permutation matching where necessary. The identification is declared stable when the grouping remains unchanged for K consecutive windows:

(40) $$Stable=I(\forall t\in \{1,\ldots ,K\},\pi_{t}=\pi_{t-1})$$

Once stability is reached, the current identification result is recorded and fixed for the ongoing acquisition. This design suppresses transient ambiguity that may occur under weak load fluctuations or highly correlated operating conditions.

Because the proposed identification relies on load-driven temporal fluctuations, the current acquisition time was set according to operating conditions but was constrained to be no less than 3 min to ensure that sufficient fluctuation information was captured; otherwise, short windows may not contain enough discriminative dynamics for reliable grouping.

### 5.3. Field Identification Results and Representation Diagnostics

**Figure 16:**
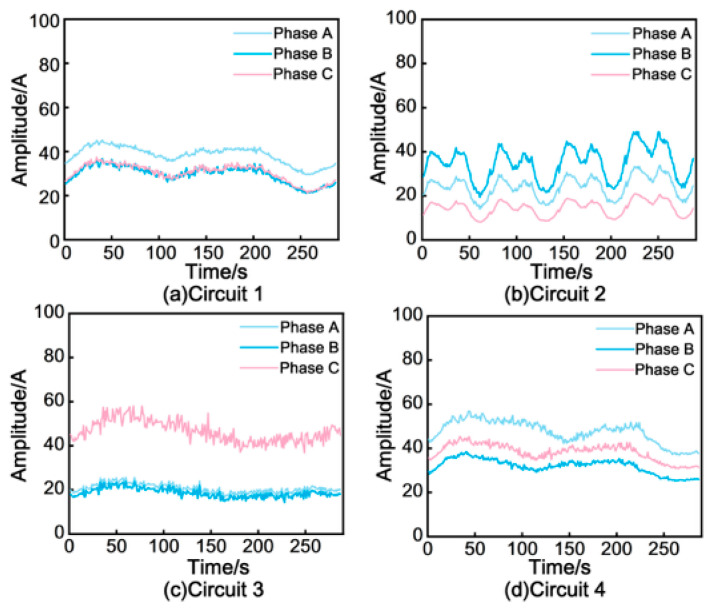
The clustering results of the four-circuit cable.

**Circuit identification results.** Figure 16 displays representative field results on a four-circuit cable system. Each sub-plot corresponds to one circuit, illustrating the sheath-current waveforms of phases A, B, and C grouped by their inferred circuit membership over the acquisition period. Observably, the clustering output successfully grouped the three-phase channels belonging to each circuit into their correct clusters. Further analysis of this result strongly validates the Temporal Similarity Criterion proposed in Section 2.2; although the sheath current amplitudes within the same circuit exhibited slight variations due to asymmetrical cable arrangement, their fluctuation trends remained highly synchronized. This observation aligns perfectly with the theoretical derivation, demonstrating that the deployed pipeline can provide stable and interpretable circuit grouping under practical conditions using only synchronous sheath-current measurements.

**Figure 17:**
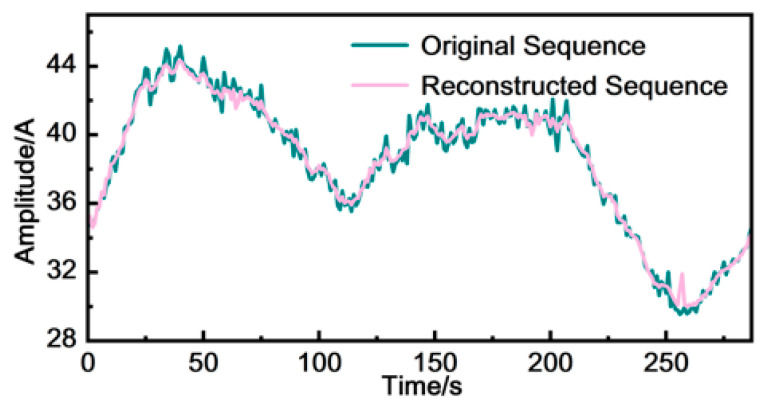
Original vs. Reconstructed Series Comparison.

**Autoencoder reconstruction as a deployment diagnostic.** In addition to the final clustering output, the reconstruction behavior of the deployed TCN-AE was inspected as a sanity check that the learned representation preserves the dominant temporal structures of sheath-current signals. Figure 17 compares the original sheath-current sequence of Phase A in the first circuit with its reconstructed sequence. The Pearson correlation coefficient of 0.98 and an MAE of 0.012 demonstrate a high degree of consistency between the two. This indicates that the encoder–decoder has learned a compact representation that retains the major waveform characteristics, which is beneficial for stable downstream clustering.

**Figure 18:**
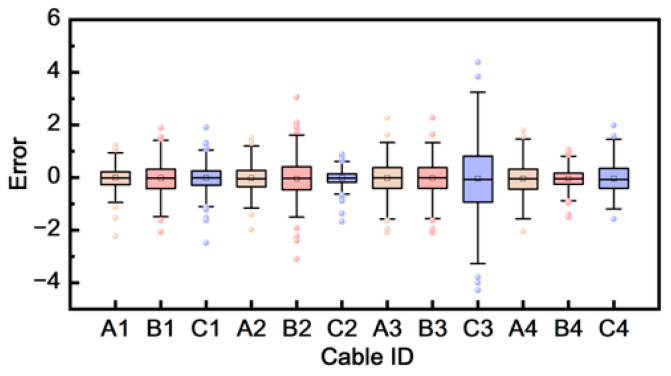
Reconstruction Error Distribution Across Cables.

**Reconstruction consistency across channels**. Figure 18 further reports the distribution of reconstruction errors across multiple cable channels using box plots. The error distributions show that reconstruction quality remains consistently controlled across different channels and configurations in the reported test, suggesting that the representation learning step does not rely on a small subset of easy channels and can generalize across multi-channel sheath-current measurements. This consistency supports the robustness of the deployed feature extraction stage prior to clustering.

### 5.4. Analysis of Maximum and Mean Deviations Between Simulation and Experiment

To evaluate whether the physics-based simulation provides a sufficiently faithful surrogate of field measurements, we conducted a distribution-level comparison between the simulation domain and the real domain. The simulation model preserved the physical topology of the experimental platform, including cable geometry, cross-bonding structure, and transmission-line parameters. Time-varying operating conditions were introduced through a slowly varying load resistance modeled as an autoregressive stochastic process, whose temporal correlation was estimated from real data. The load variation was constrained within a realistic engineering interval. In addition, mild measurement-chain effects were incorporated, including small gain and bias perturbations, low-frequency drift, colored noise, and weak inter-channel coupling, to better reflect practical acquisition characteristics. Under this design, the simulated waveforms exhibited non-trivial temporal variability and avoided being overly clean or trivially aligned with real measurements; nevertheless, some residual simulation-to-real discrepancy is still expected due to inevitable modeling simplifications and unmodeled environmental factors.

To quantify the domain discrepancy, the squared Maximum Mean Discrepancy (MMD) was computed using the unbiased finite-sample estimator:

(41) $${\mathrm{MMD}}_{u}^{2}=\frac{1}{n(n-1)}\sum_{i\neq j}k(x_{i},x_{j})+\frac{1}{m(m-1)}\sum_{i\neq j}k(y_{i},y_{j})-\frac{2}{nm}\sum_{i,j}k(x_{i},y_{j})$$

where *x_i_* denotes real samples and *y_j_* denotes simulated samples. For MMD, each channel time series is represented by a fixed-dimensional handcrafted summary feature vector (statistical moments, variability, differential dynamics, auto correlation, and spectral energy). We compared 12 real samples with 120 simulated samples. The test yielded a biased MMD^2^ = 0.233159, unbiased MMD^2^ = 0.172975, a kernel bandwidth σ = 3.372703, and permutation *p* = 0.001, indicating a statistically significant shift, as idealized mathematical analytical models fundamentally cannot perfectly replicate the complex variations of real-world environments.

This shift is an expected consequence of inevitable modeling simplifications and unmodeled environmental factors. However, the proposed approach remains effective because the discriminative structure it exploits is governed by fundamental electromagnetic constraints rather than purely statistical similarity. Since the cable system is a coupled transmission-line network governed by Maxwell’s equations, the intrinsic geometric and coupling relationships among phases are preserved across both domains. Consequently, moderate distribution shifts in marginal statistics may alter waveform appearance but do not destroy the underlying coupling topology that defines cluster separability in the learned latent space.

### 5.5. Practical Considerations, Boundary Conditions, and Limitations

The field deployment confirmed the feasibility of online circuit identification using sheath-current time-series patterns in a learned representation space. Two practical boundary conditions should be emphasized. First, under extremely low load conditions, the induced component becomes weak and the effective SNR decreases, which may increase short-term ambiguity; in such cases, longer acquisition windows and stricter stability requirements such as larger W or K are recommended. Second, when two circuits operate under highly correlated load fluctuations, also known as same-load mode, inter-circuit similarity can increase, narrowing the separation margin; therefore, stable decisions should rely on longer observation and the stability criterion rather than instantaneous clustering outputs.

Finally, this section focused on the normal operating conditions of the test platform. It is worth noting that for non-cross-bonded cables, including single-point bonding or solid bonding at both ends, their distinct current magnitudes allow for efficient identification via pre-screening, enabling the deep clustering model to focus on the complex cross-bonded scenarios. In practical systems, defects or abnormal states in cross-bonding grounding systems may introduce anomalies in sheath-current time-series patterns and affect clustering reliability. This motivates the further investigation of defect-affected scenarios and robustness enhancement strategies, such as anomaly-aware preprocessing or joint identification-and-diagnosis frameworks. Furthermore, the field validation is fundamentally limited to qualitative feasibility proof due to the physical difficulty of acquiring objective ground-truth labels for absolute quantitative evaluation. This explicitly highlights the necessity of integrating unsupervised domain adaptation techniques in future research to bridge the statistical simulation-to-reality gap.

## 6. Conclusions

To address HV cable circuit online identification challenges arising from missing or degraded cable nameplates, an online circuit identification method for cross-bonded cable systems based on deep embedded clustering was proposed. Time-series samples were generated using a multi-circuit sheath-current calculation model for network training, and the effectiveness of the proposed method was validated through both simulation studies and full-scale cable platform experiments. The main conclusions can be summarized as follows:

(1) A TCN-AE-based cable circuit identification model was developed with optimized hyperparameters and network structure, achieving an accuracy of 95.37%. Representation diagnostics confirm that compared to the raw signal space, this nonlinear deep feature extraction significantly reduced the dispersion ratio of the latent features, demonstrating clear advantages over baseline methods
(2) When the minor-section lengths of cross-bonded cables were identical, the induced components of sheath currents within the same circuit exhibited strong temporal consistency. DTW-based evaluations demonstrated that intra-circuit similarity remained higher than inter-circuit similarity across different system parameters and operating conditions, supporting temporal similarity as a reliable circuit identification criterion.
(3) Simulation and experimental results further confirmed that the proposed method does not require direct load-current measurement and maintains stable identification performance under variations in minor-section length and cable configuration modes; these validate that the model remains highly robust against the simulation-to-reality domain gap.

It should be noted that cable defects may introduce anomalies in sheath-current time-series data, which could affect the identification accuracy for circuits containing defective cables. Future work will focus on investigating the influence of defect-induced temporal distortions and incorporating defect-aware modeling strategies to further enhance the robustness and generalization capability of the proposed method. Additionally, integrating unsupervised domain adaptation architectures will be a primary focus to systematically bridge the quantitative validation gap between simulation models and real-world engineering environments.

## Figures and Tables

**Table 1:** Similarity-based criteria for circuit identification.

Similarity Level	Similarity Coefficient Range(S)	Circuit Relationship
High Similarity	0.7~1.0	Same Circuit
Transition Zone	0.4~0.7	Uncertain
Low Similarity	0.0~0.4	Different Circuit

**Table 2:** Cable simulation parameters.

Cable Parameter	Value	Cable Parameter	Value
Core outer diameter/mm	38.9	Sheath resistivity/Ω·m	2.8 × 10^−8^
Insulation outer diameter/mm	70.9	Grounding resistance/Ω·m	0.5
Sheath outer diameter/mm	99.6	Operating phase voltage/kV	63.5
Sheath inner diameter/mm	97.3	XLPE relative permittivity	2.3
Core resistivity/Ω·m	1.68 × 10^−8^	Soil resistivity/Ω·m	100

**Table 3:** Unsupervised clustering quality metrics.

ARI	NMI	DBI	Silhouette
0.581	0.665	0.303	0.865

**Table 4:** Comparison results.

Algorithm	Accuracy/%	Precision/%	Recall/%	F1/%
TAE-K-medoids	95.37	95.53	95.37	95.39
TAE-K-means	92.27	92.56	92.27	92.29
LAE-K-medoids	86.57	87.09	86.57	86.47
K-medoids	83.52	85.17	83.52	82.86

## Data Availability

The data used to support the findings of this study are available from the corresponding author upon reasonable request.
